# Supplementary material for: Dynamic structure of E. coli cytoplasm: supramolecular complexes and cell aging impact spatial distribution and mobility of proteins
Source: Commun Biol. 2024 Apr 27;7:508. doi: 10.1038/s42003-024-06216-3 (PMC11055878; doi:10.1038/s42003-024-06216-3)
Supplement: Supplementary file 2 — Supplementary Information [file 42003_2024_6216_MOESM2_ESM.pdf]

Supplementary Information

Dynamic structure of *E. coli* cytoplasm: supramolecular complexes and cell aging impact spatial distribution and mobility of proteins.

Dmitrii Linnik, Ivan Maslov, Christiaan Michiel Punter and Bert Poolman*

Department of Biochemistry, University of Groningen

Groningen, Nijenborgh 4, 9747 AG, the Netherlands.

*E.mail: b.poolman@rug.nl

Keywords: spatial organization of bacterial cytoplasm, lateral diffusion, protein aggregation and condensates, bacterial cell ageing, single-molecule diffusion

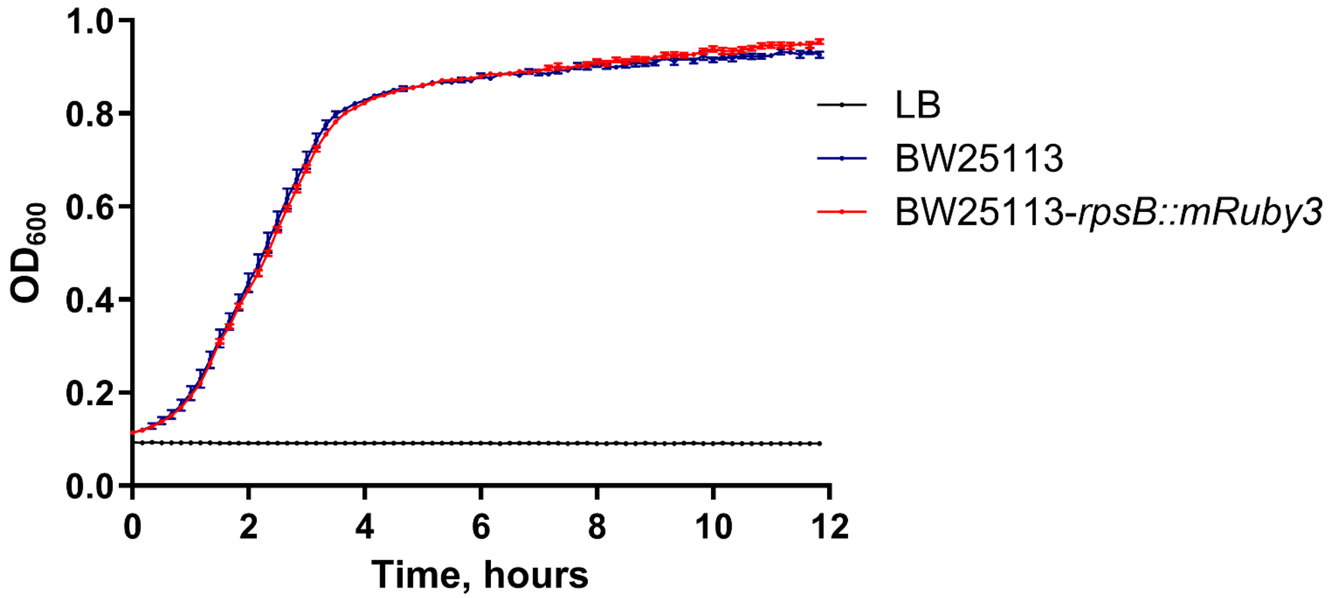

Fig. S1. Growth curves of *E. coli* BW25113 and BW25113-*rpsB::mRuby3* (with chromosomal integration of the *mRuby3* gene 3' end of the *rpsB* gene). Data presented as Mean ± SEM and Number of repeats is 5. LB is Luria-Broth without inoculation.

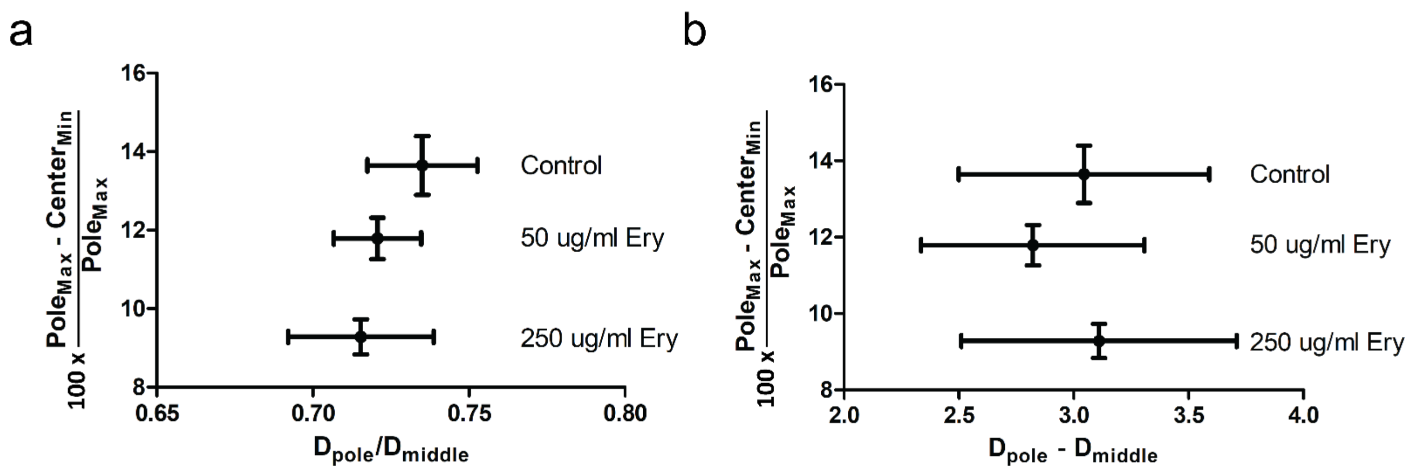

Fig. S2. The ribosomal distribution profile does not correlate with the difference between diffusion coefficient at the center and poles of erythromycin-treated *E. coli*. The dependence of 100 × ($PoleMax{-}CenterMin$) / $PoleMax$ on the ratio of pole/middle lateral diffusion **(a)** and on the difference between D_{pole} and D_{middle} **(b) are** not significant; the Spearman's rank correlation coefficient, p-values are 0.33 for **(a)** and 1.00 for **(b)**.

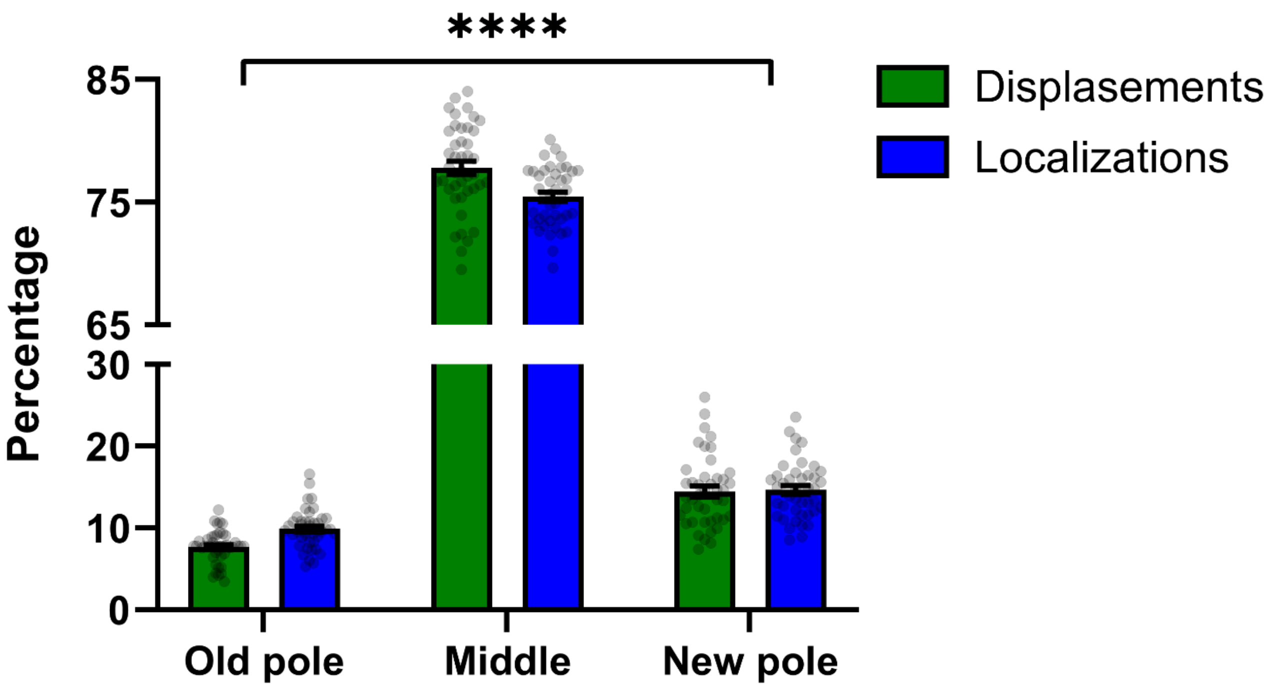

Fig. S3. Data distribution of mEos3.2 displacements and localizations in *E. coli* BW25113-*mEos3.2* cells; the data are presented as percentage of all observed displacements (green) or localizations (blue). The data distribution is significantly different for the old and new pole of dividing cells. Data presented as mean ± SEM; the number of dividing BW25113-*mEos3.2* cells is 43. The significance level is presented as asterisk signs: (****) for p<0.0001.

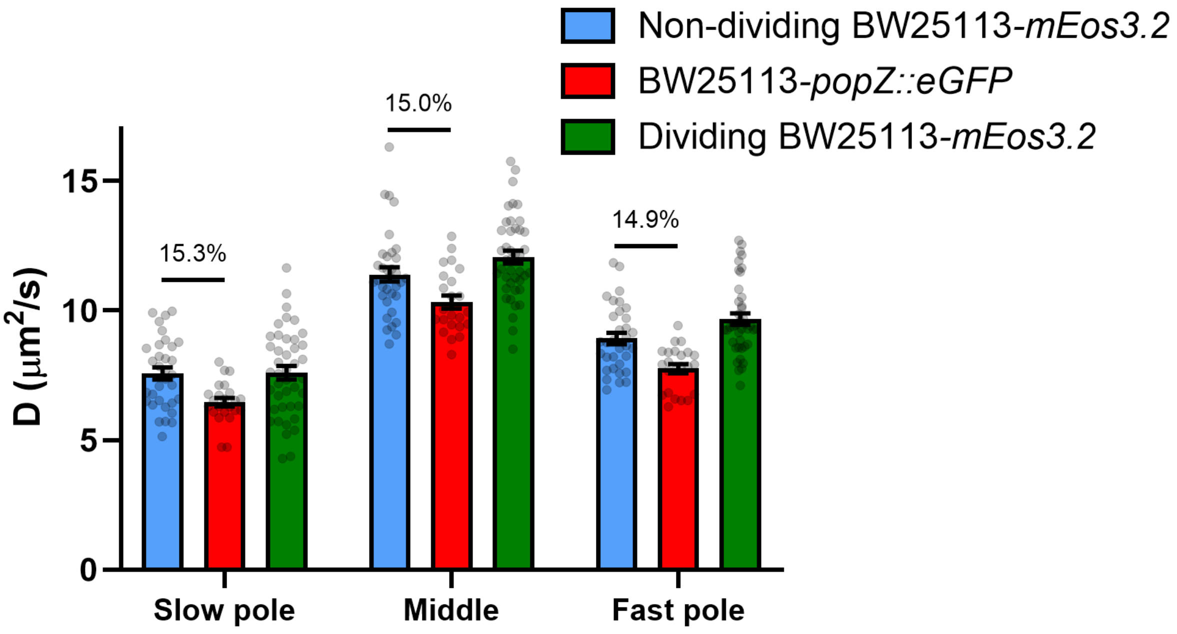

Fig. S4. Region-unspecific decrease of mEos3.2 D_L in *E. coli* BW25113 cells overexpressing PopZ. A decrease of ~ 15% in mEos3.2 mobility is observed both in the middle part of the cells, and the fast and slow pole, compared to both dividing and non-diving BW25113-*mEos3.2*. Data presented as mean ± SEM; the number of nondividing BW25113-*mEos3.2*, dividing BW25113-*mEos3.2* and BW25113-*popZ::eGFP* cells is 35, 39 and 23, respectively.

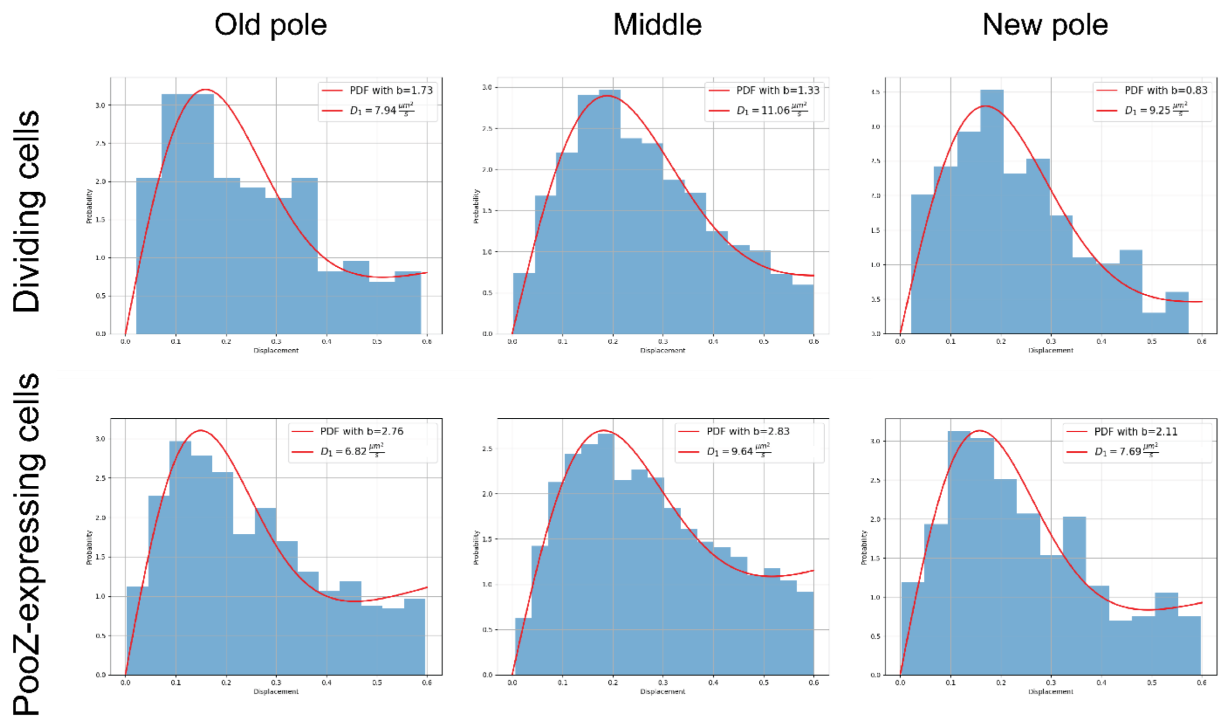

Fig. S5. Representative probability density function fitting profiles of the displacements in the middle part old pole and new pole of dividing *E. coli* BW25113-*mEos3.2* and BW25113-*popZ::eGFP*, expressing PopZ-eGFP as a maker of the old pole. Equation (3), using maximum likelihood estimation, was used to fit the data. D_1 and b refer to lateral diffusion coefficient (D_L) and background correction coefficient, respectively.

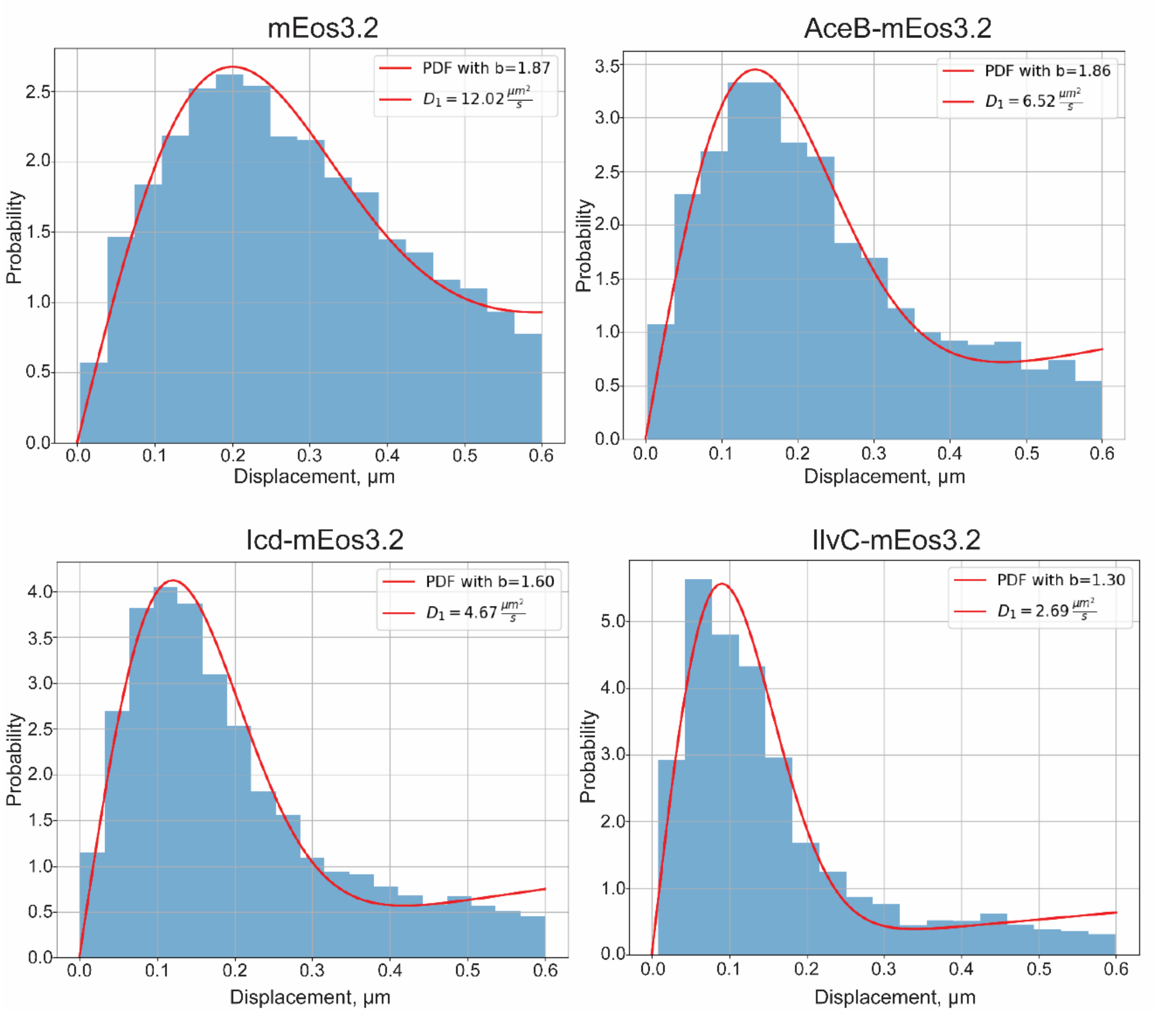

Fig. S6. Representative probability density function fitting profiles of the displacements in the middle part of *E. coli* BW25113, expressing mEos3.2, AceB-mEos3.2, Icd-mEos3.2 or IlvC-mEos3.2. Equation (3), using maximum likelihood estimation, was used to fit the data. D_1 and b refer to lateral diffusion coefficient (D_L) and background correction coefficient, respectively.

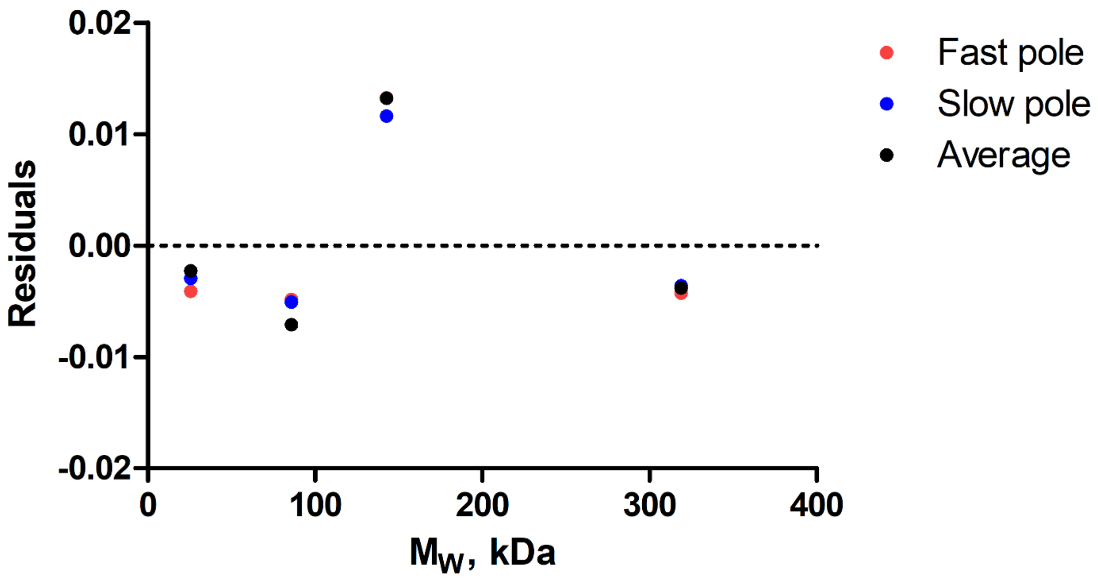

Fig. S7. Analysis of the residuals from the plots shown in Figure 4D. The analysis shows no signs of correlation for the residuals.

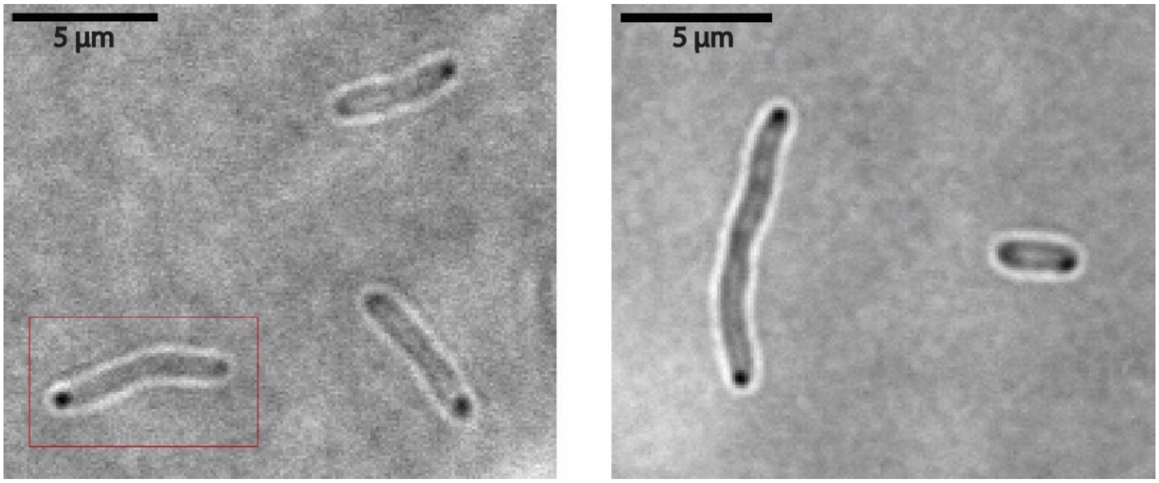

Fig. S8a. Brightfield images of *E. coli* BW25113-*mEos3.2* cells treated with cephalexin for 4h, followed by a 1h heat-shock at 42°C before diffusion measurement to induce protein aggregation. The cell in the box was used in figure 7C.

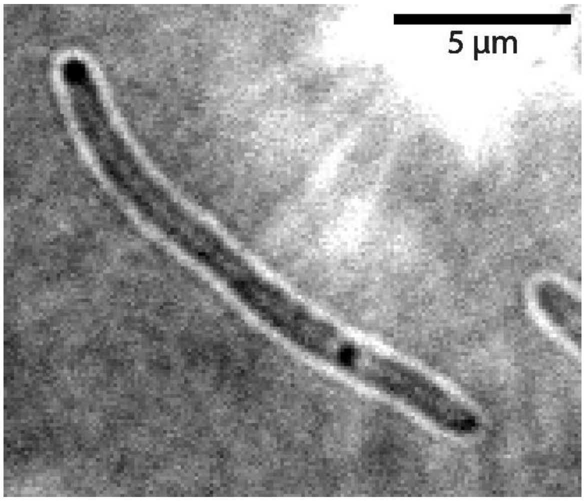

Fig. S8b. Brightfield image of *E. coli* BW25113-*mEos3.2* cell used in figure 7C, treated with cephalexin for 7h, followed by a 1h heat-shock at 42°C before diffusion measurement to induce protein aggregation.

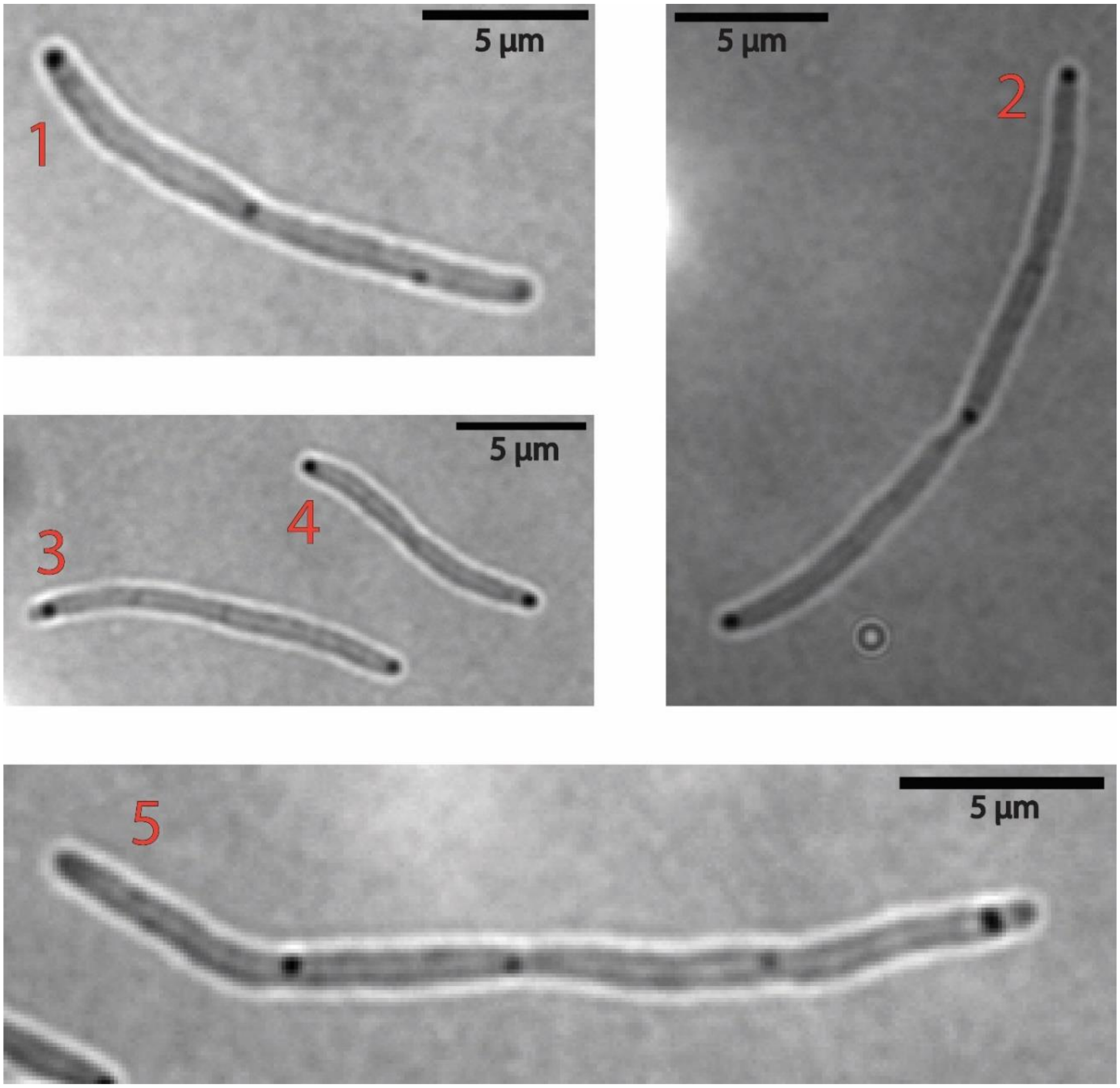

Fig. S9. Brightfield images of *E. coli* BW25113-*mEos3.2* cells treated with cephalexin for 7h, followed by a 1h heat-shock at 42°C before diffusion measurement to induce protein aggregation. The numbers correspond to the diffusion and displacement maps of figure S10.

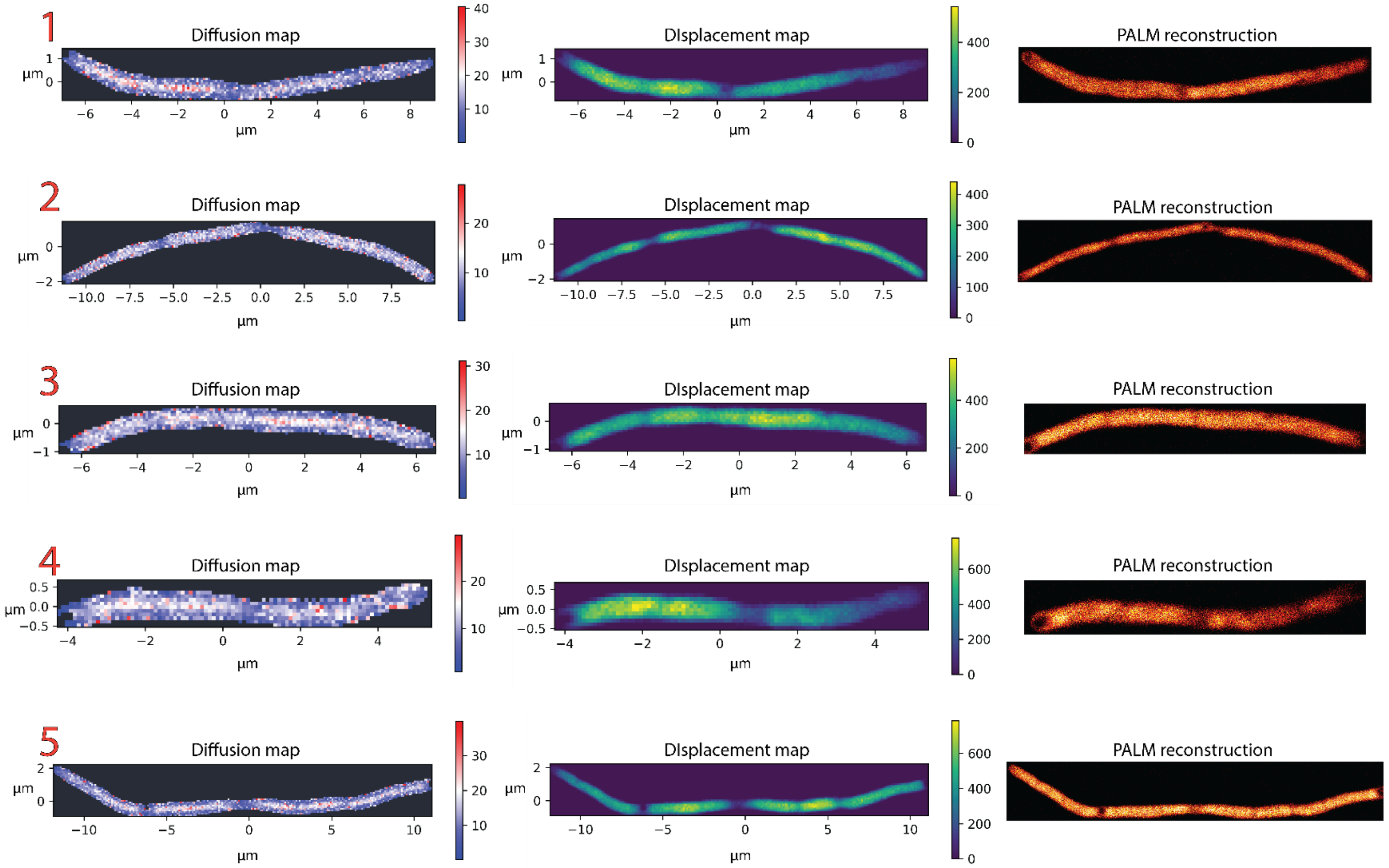

Fig. S10. Displacement, diffusion maps and PALM reconstructions of *E. coli* BW25113-*mEos3.2* cells treated for 7h with cephalexin followed by a 1h heat-shock as described in the legend of Figure S9. The pixel bin size of the displacement and diffusion maps is 100 nm. Color map for displacement maps represents the number of displacements per pixel. Diffusion map reconstructed by fitting displacements in each pixel bin with equation (3). The numbers refer to the brightfield images shown in Figure S9.

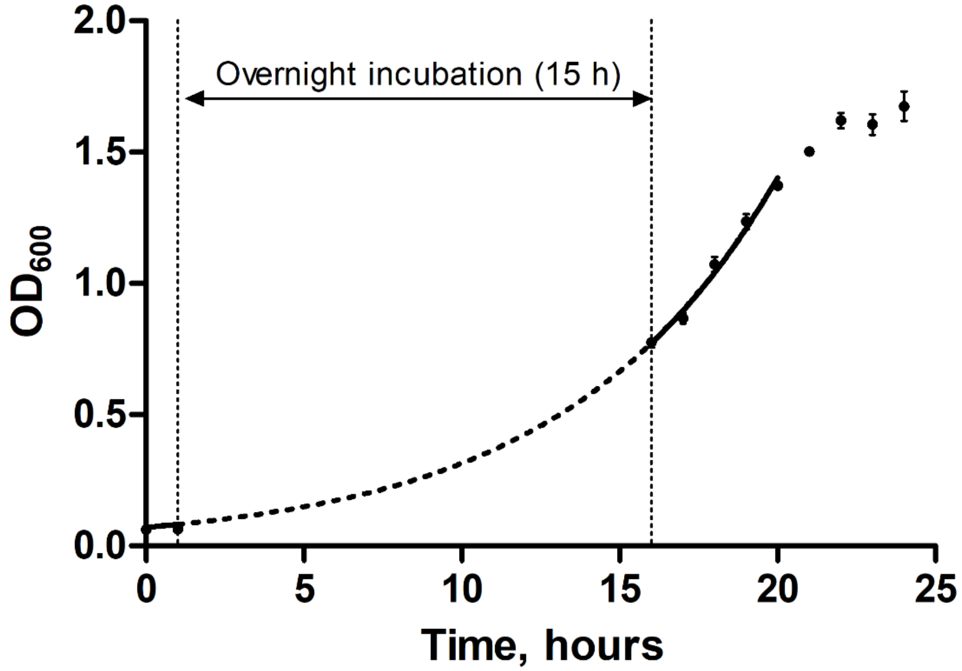

Fig. S11. Growth of *E. coli* BW25113-*mEos3.2* strain before and after overnight incubation in MBM media supplemented with 0.1% (v/v) glycerol at 30°C. After 15-hour overnight incubation in a time period of 1-2 hours cells were diluted to OD_{600} ~0.05 and grown for microscopy analysis.

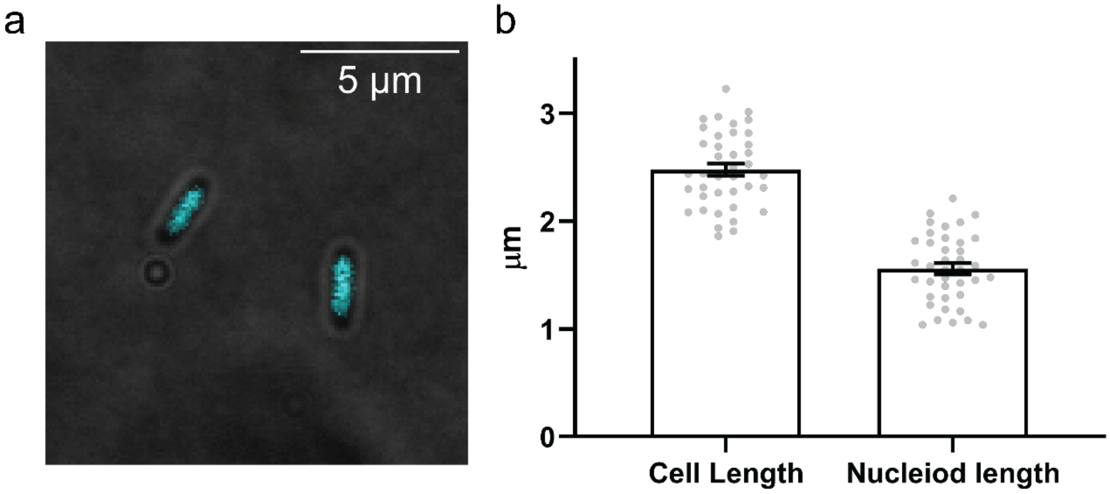

Fig. S12. Nucleoid staining of *E. coli* BW25113-*mEos3.2*. **(a)** Representative overlay of brightfield (grey scale) and fluorescent (cyan) images of cells stained with 15 µM DAPI. The 405 nm laser was used for excitation and the emitted light was collected in the spectral range from 500 to 550 nm, using a ET 525/50 M bypass filter (Chroma). **(b)** Length of analyzed cells and nucleoid-occupied areas along the long axis of the cell. The nucleoid occupies 63±1% of the cell length; the cell length was 2.47±0.06 µm and nucleoid length was 1.56±0.05 µm. Data presented as a mean value ± standard error of the mean (SEM).

Table S1. Primers used in this work.

Primer name	Used as	5' to 3' sequence
eGFP _for	Forward primer to amplify *eGFP* gene	ACCGGTGGTAGCUCTAAAGGTGAAGAATTATTC
eGFP _rev	Reverse primer to amplify *eGFP* gene	ATTATTTGUACAATTCATCCATACC
PopZ_for	Forward primer to amplify *popZ* gene	ACAAATAAUAAAAGCTTCTGTTTTGGCGGATGAG
PopZ_rev	Reverse primer to amplify *popZ* gene	AGCTACCACCGGUGCCACC
pZ8 -Ptac _for	Forward primer to amplify pZ8-Ptac vector (segment 1)	AGGTGGTGGUAAGCTTCTGTTTTGGCGGATGAG
pZ8 -Ptac _rev	Reverse primer to amplify pZ8-Ptac vector (segment 1)	ATCGCTCAUGAATTCTGTTTCCTGTGTGAAATTGTTATCC
pZ8-Ptac-KanR _for	Forward primer to amplify pZ8-Ptac vector (segment 2)	ATTCCGACTCGCCAACATCAATACA
pZ8-Ptac-KanR _rev	Reverse primer to amplify pZ8-Ptac vector (segment 2)	ACGAGTCGGAACGCAGACC
