# Supplementary material for: Dynamic structure of E. coli cytoplasm: supramolecular complexes and cell aging impact spatial distribution and mobility of proteins
Source: Commun Biol. 2024 Apr 27;7:508. doi: 10.1038/s42003-024-06216-3 (PMC11055878; doi:10.1038/s42003-024-06216-3)
Supplement: Supplementary file 3 — Description of Additional Supplementary Files [file 42003_2024_6216_MOESM3_ESM.pdf]

Description of Additional Supplementary Files

File name: Supplementary Data 1

Description: The source data behind the graphs in this paper
